# The Québec BCG Vaccination Registry (1956–1992): assessing data quality and linkage with administrative health databases

**DOI:** 10.1186/1472-6947-14-2

**Published:** 2014-01-09

**Authors:** Marie-Claude Rousseau, Florence Conus, Jun Li, Marie-Élise Parent, Mariam El-Zein

**Affiliations:** 1 Epidemiology and Biostatistics Unit, INRS-Institut Armand-Frappier, Université du Québec, 531 boul. des Prairies, Laval, QC H7V 1B7, Canada

**Keywords:** Bacillus Calmette-Guérin, Administrative databases, Registry, Validity, Linkage, Epidemiology

## Abstract

**Background:**

Vaccination registries have undoubtedly proven useful for estimating vaccination coverage as well as examining vaccine safety and effectiveness. However, their use for population health research is often limited. The Bacillus Calmette-Guérin (BCG) Vaccination Registry for the Canadian province of Québec comprises some 4 million vaccination records (1926-1992). This registry represents a unique opportunity to study potential associations between BCG vaccination and various health outcomes. So far, such studies have been hampered by the absence of a computerized version of the registry. We determined the completeness and accuracy of the recently computerized BCG Vaccination Registry, as well as examined its linkability with demographic and administrative medical databases.

**Methods:**

Two systematically selected verification samples, each representing ~0.1% of the registry, were used to ascertain accuracy and completeness of the electronic BCG Vaccination Registry. Agreement between the paper [listings (n = 4,987 records) and vaccination certificates (n = 4,709 records)] and electronic formats was determined along several nominal and BCG-related variables. Linkage feasibility with the Birth Registry (probabilistic approach) and provincial Healthcare Registration File (deterministic approach) was examined using nominal identifiers for a random sample of 3,500 individuals born from 1961 to 1974 and BCG vaccinated between 1970 and 1974.

**Results:**

Exact agreement was observed for 99.6% and 81.5% of records upon comparing, respectively, the paper listings and vaccination certificates to their corresponding computerized records. The proportion of successful linkage was 77% with the Birth Registry, 70% with the Healthcare Registration File, 57% with both, and varied by birth year.

**Conclusions:**

Computerization of this Registry yielded excellent results. The registry was complete and accurate, and linkage with administrative databases was highly feasible. This study represents the first step towards assembling large scale population-based epidemiological studies which will enable filling important knowledge gaps on the potential health effects of early life non-specific stimulation of the immune function, as resulting from BCG vaccination.

## Background

Several country-specific experiences with established population-based vaccine registries have demonstrated the utility of vaccination registries for surveillance purposes and management of vaccination programmes [1,2]. Indeed, these registries have proven useful for estimating vaccination coverage [3-11], examining vaccine safety and effectiveness [3-6,8,10], enabling identification of poorly immunised at-risk population subgroups [7], as well as identifying determinants of vaccination and addressing research hypotheses on certain vaccine-disease relations [3,4]. A broader use of these registries for research purposes has also been proposed [1,2]. However, in order to exploit their full potential for population health research, vaccination registries need to be in an electronic format and linkable with medical databases. This allows, for instance, a long term follow-up of vaccinated and non-vaccinated individuals for the occurrence of medical conditions of interest.

In an effort to prevent tuberculosis, an organized voluntary program of vaccination with Bacillus Calmette-Guérin (BCG) was held in the province of Québec, Canada, from 1949 to 1974. The vaccine was mostly offered to newborns and schoolchildren. Before vaccination, all except newborns were subjected to a tuberculin reactivity test. All vaccination certificates – on which were recorded tuberculin reactivity tests and BCG vaccinations – were sent to a centralized registry. The Québec BCG Vaccination Registry, presently kept at INRS (*Institut national de la recherche scientifique*)-Institut Armand-Frappier, comprises some 4 million entries (1926–1992). The first immunizations with BCG in Québec occurred in 1926, but routine use began in 1949. Before 1949, BCG vaccination was mainly used for research purposes, including testing the safety and efficacy of the vaccine. Until recently, this registry was organized into searchable microfilms (1926–1955) and paper listings (1956–1992) produced from original vaccination certificates. Its entire content has recently been digitized.

With a view to using the electronic Québec BCG Vaccination Registry in large scale population-based epidemiological studies, we firstly intended to assess its data quality, and secondly to examine its linkability with administrative medical and demographic databases.

## Methods

### Organization of the Québec BCG Vaccination Registry

Vaccination certificates covering the years between 1926 and 1955 inclusively were only available on microfilms. Beginning in the 1960s, clerical staff manually entered the information from vaccination certificates dated 1956 onwards to create listings for consultation purposes. Eight series of alphabetically sorted paper listings were produced, each covering a few calendar years from 1956 to 1992. This represented 140 volumes totalling over 60,000 pages (~4.2 million lines of data), which, along with 123 microfilm rolls, constituted the registry’s searchable hard-copy. In the paper listings, information corresponding to an individual event was summarized on one line that specifically contained information on surname and given name, father’s given name, birth date, sex, tuberculin reactivity test (type, date, and reaction in millimetres), current vaccination (date and administration mode), date of prior vaccinations, and medical institution code.

Vaccination certificates (microfilms and paper) and paper-based listings of the BCG Vaccination Registry have been converted to an electronic format in 2010. Computerization, performed by Trigonix Inc. (Montreal, Canada), comprised graphical imaging of vaccination certificates (microfilms and paper) and transfer of paper listings into a searchable electronic database using optical character recognition. In a first step, this involved scanning each page within the registry’s paper listings to create PDF (Portable Document Format) files. In a second step, the optical character recognition parameters were optimized for each of the eight series of paper listings due to their differing formats, thereby allowing their conversions into an electronic database.

### Data quality

The quality and accuracy of the electronic version of the Québec BCG Vaccination Registry, hereafter referred to as the electronic BCG Registry, was ascertained using two distinct verification samples. Sample size was determined based on having a feasible number of records to retrieve and review, and achieving a reasonable statistical precision. Firstly, we determined agreement between paper listings and the electronic BCG Registry to assess accuracy of the computerization process. Individual records (n = 5,268) from paper listings (1956–1992) were sampled by systematically selecting pages within volumes, and lines within pages to roughly represent 0.1% from each of the eight series. Secondly, we determined agreement between vaccination certificates and the electronic BCG Registry to document the accuracy and completeness of the electronic database compared with its archived raw data. Vaccination certificates, stored in archive filing cabinets, are organized by year and geographical region where vaccination took place. We systematically selected 4,972 vaccination certificates (~0.1% of the registry), approximately 250 certificates for each of the 20 years of interest (1956–1975) sampled from all available geographical regions.

For the two samples, the quality control and verification process was documented in terms of the completeness of information and presence of discrepancies. Information was compared along several personal nominal identifiers and BCG-related variables. We computed, for each variable, percent agreement and 95% confidence intervals (CIs) among records containing valid information (non-empty fields). The proportion of records with complete agreement on all variables was also calculated. Verification and analyses were respectively carried out with Filemaker Pro, version 11.0 (FileMaker Inc., Santa Clara, California) and SPSS, version 17.0 (SPSS Inc., Chicago, Illinois).

### Linkage with administrative databases

To assess linkage feasibility, we determined the proportion of successful record linkages with provincial demographic and administrative medical databases, overall and per birth year. From the electronic BCG Registry, 3,500 subjects were randomly sampled among 491,861 individuals born from 1961 to 1974 (~250 per birth year) and vaccinated between 1970 and 1974. The nominal information extracted included child’s surname, given names of the child and father, sex, and date of birth. Data linkage was independently performed between the electronic BCG Registry and both the Birth Registry, administered by the *Institut de la statistique du Québec* (ISQ), and the Healthcare Registration File (*Régie de l’assurance maladie du Québec*, RAMQ). The Birth Registry provides information on all births and stillbirths occurring in the province as well as perinatal and parental sociodemographic data, retrieved from birth certificates. The RAMQ is the government body responsible for administration of health care services in Québec. Our linkage was conducted with the Healthcare Registration File which includes, for all beneficiaries of the universal public health system, information such as birth date, sex, postal code and year of death (if applicable).

Probabilistic record linkages between the electronic BCG Registry and the Birth Registry were done by the *Environnement pour la promotion de la santé et du bien-être* (EPSEBE) team at ISQ [12] using five basic identifiers (child’s surname and given name, date of birth, sex, initial of father’s given name). Deterministic linkages between the electronic BCG Registry and the Healthcare Registration File were carried out by RAMQ using the same identifiers, except for using the full father’s given name instead of his initial. For matching purposes, nominal information was independently standardized by ISQ and RAMQ in several ways (e.g., capitalizing all letters, eliminating general identifiers such as “Ms.” or “unknown”, standardizing date formats, removing blank spaces, splitting hyphenated names into several data fields). This entire project, including the transfer of confidential data, was done in accordance with the province of Québec’s legal and ethical requirements. Procedures, data access, and ethical issues were assessed and approved by the *Commission d’accès à l’information* (reference number: 11 02 67 (10 08 48, 09 08 39)), INRS Research Ethics Committee (reference number: CER-09-203), and ISQ (reference number: KB2-Rousseau-pilote, 09–08).

The presence of certain potential confounders pertinent to investigating hypotheses on a link between BCG vaccination and chronic diseases was verified, given that a typical limitation of using administrative databases for research purposes is the lack of information on such variables. Some useful variables documented in the Birth Registry over the years covered include gestational age, birth weight, number of older siblings, mother’s municipality of residence, as well as parents’ age and birthplace.

## Results

Table 1 presents, for both verification samples, the number of discrepancies and proportion of agreement per variable for “non-empty” fields among those verified. Out of 5,268 records selected from paper listings, 281 were duplicates. For each of those, only one entry was kept for verification. As such, the first verification sample comparing paper listings to the electronic BCG Registry comprised 4,987 records. Overall, a very high agreement per variable was observed, ranging from 99.8% to 100%. There were 20 records with a maximum of one discrepancy observed among the 13 variables considered. This represented perfect agreement for 99.6% of verified records. Of these 20 discrepancies, 6 (30%) were in text fields and 14 (70%) in numeric fields. Errors mainly occurred during optical character recognition (n = 16) and, to a lesser extent, data validation following computerization (n = 4).

**Table 1:** Data quality ascertainment of the electronic BCG Registry, expressed as proportion of agreement per variable

	**Paper listings vs. electronic BCG Registry (1956–1992)**			**Vaccination certificates vs. electronic BCG Registry (1956–1975)**		
	**N = 4,987**			**N = 4,709**		
	**N**^**a**^	**No. of Discrepancies**	**% Agreement (95% CI)**	**N**^**b**^	**No. of Discrepancies**	**% Agreement (95% CI)**
**Nominal variables**						
Surname	4986	0	100.0 (-)	4704	23	99.5 (99.3-99.7)
Given Name	4772	3	99.9 (99.8-100.0)	4639	364	92.2 (91.3-92.9)
Sex	4952	1	100.0 (99.9-100.0)	4697	393	91.6 (90.8-92.4)
Date of Birth	4921	4	99.9 (99.8-100.0)	4702	25	99.5 (99.2-99.6)
Father’s Given Name	4889	2	100.0 (99.9-100.0)	4630	54	98.8 (98.5-99.1)
**BCG-related variables**						
Tuberculin reactivity test						
Year	3754	0	100.0 (-)	2962	1	100.0 (99.8-100.0)
Type	3754	0	100.0 (-)	2962	1	100.0 (99.8-100.0)
Reaction	3478	2	99.9 (99.8-100.0)	2818	33	98.8 (98.4-99.2)
Vaccination						
Year	3369	0	100.0 (-)	3790	0	100.0 (-)
Type	1965	0	100.0 (-)	3541	7	99.8 (99.6-99.9)
Institution	4963	8	99.8 (99.7-99.9)	-	-	-
Year of 1^st^ prior vaccination	637	0	100.0 (-)	407	3	99.3 (97.9-99.7)
Year of 2^nd^ prior vaccination	402	0	100.0 (-)	243	4	98.4 (95.8-99.4)

^a^ Number of records with a non-empty field, for each variable, among 4,987 verified records.

^b^ Number of records with a non-empty field, for each variable, among 4,709 verified records.

With respect to data quality ascertainment in the second verification sample, 4,709 (94.7%) out of 4,972 vaccination certificates were found in the electronic BCG Registry. The proportion of exact agreement per variable was high. It was lowest for sex and given name (91.6% and 92.2%, respectively), and highest (100%) for year and type of tuberculin reactivity test as well as the year of BCG vaccination. Compared with their corresponding vaccination certificates, exact agreement was observed on all 12 variables for 3,840 (81.5%) records, with 1 discrepancy in 834 (17.7%) records, 2 discrepancies in 31 (0.7%) records, and 3 discrepancies in 4 (0.1%) records. A total of 908 discrepancies were found, largely in given name and sex. As shown by the error breakdown in Table 2, the most recurrent discrepancy pattern observed was having information in the electronic BCG Registry with empty fields on equivalent vaccination certificates. Such discrepancies might be attributed to data entry clerks filling in incomplete information when generating paper listings. For instance, among individuals vaccinated very shortly after birth and without a given name on vaccination certificates, generic given names were assigned instead of leaving the field blank. These included a given name commonly used in the catholic French-speaking population in Québec (i.e., “Marie” for girls and “Joseph” for boys), “unknown” or “baby”. Discrepancies sometimes resulted from truncated names due to field length limitations in paper listings. In addition, whenever an empty field for sex was found on vaccination certificates, a decision on sex classification was most probably based on the given name. No specific pattern emerged for discrepancies in variables other than given name and sex. These appeared to be the result of typographical errors during data entry or computerization.

**Table 2:** Description of discrepancies for given name and sex, comparing vaccination certificates to the electronic BCG Registry

	**Discrepancies N (%)**	**Information on vaccination certificates**	**Information in electronic BCG Registry**
**Given name**	153 (42.0)	Empty	“Marie”
(n = 364)	137 (37.6)	Empty	“Joseph”
	21 (5.8)	Full name	Truncated name
	20 (5.5)	“Baby”	Empty
	13 (3.6)	Empty	“Unknown”
	20 (5.5)	Other	Other
**Sex**	196 (49.9)	Empty	“Male”
(n = 393)	182 (46.3)	Empty	“Female”
	15 (3.8)	Other	Other

**Figure 1:**
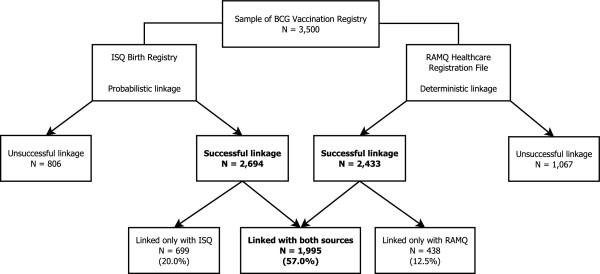
**Linkage between the electronic BCG Registry and both the Birth Registry and Healthcare Registration File.** Among 3,500 individuals born from 1961 to 1974 and vaccinated with BCG (Bacillus Calmette-Guérin) between 1970 and 1974, the linkage process identified 2,694 (77%) individuals who were linked with the ISQ (*Institut de la statistique du Québec*) Birth Registry using a probabilistic linkage method and 2,433 (70%) individuals linked with the RAMQ (*Régie de l’assurance maladie du Québec*) Healthcare Registration File using a deterministic linkage method. Linkage between the electronic BCG Registry and both databases was obtained for 1,995 (57%) individuals.

**Figure 2:**
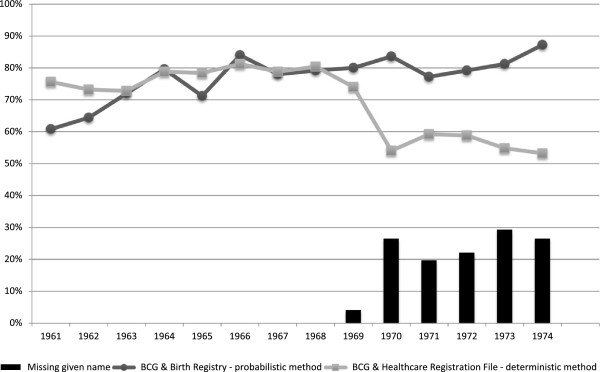
**Proportion of successful linkage by birth year.** Line connecting circular points represents the proportion of successful linkage between the electronic BCG Registry and the Birth Registry using a probabilistic record linkage. Line connecting square-shaped points represents the proportion of successful linkage between the electronic BCG Registry and the Healthcare Registration File using a deterministic record linkage. Bars represent the proportion of missing given names.

Figure 1 summarizes the data linkage process and overall proportions of record linkages. Out of 3,500 records, 89.5% were successfully linked with either the Birth Registry or the Healthcare Registration File. The majority (57%) were linked with both, whereas 20% and 12.5% were linked exclusively with the former and the latter, respectively. Figure 2 illustrates, by birth year, the proportion of records from the electronic BCG Registry successfully linked with the Birth Registry and the Healthcare Registration File, and of missing given names. The proportion of successful linkage varied by birth year, data source and linkage method. Generally, better linkage was achieved with the Birth Registry than the Healthcare Registration File, except for certain years (60.8% vs. 75.6% in 1961; 64.4% vs. 73.2% in 1962; and 71.2% vs. 78.4% in 1965). Among the identification variables used for linkage, given name had the highest proportion of missing values (4.3%). This proportion was high (20-30%) among those born and vaccinated between 1970 and 1974, mainly due to newborns for whom no given name had been assigned at the time of vaccination. Consequently, among those born from 1970 to 1974, much higher proportions of successful linkage of the electronic BCG Registry were achieved with the Birth Registry through probabilistic linkage, than with the Healthcare Registration File applying a deterministic approach. As a sensitivity analysis, the proportions of linkage among those born from 1969 to 1974 were calculated after excluding subjects with a missing given name (none among those born between 1961 and 1968). These proportions were substantially higher than when considering all subjects. They were on average 6.3% higher for linkage with the Birth Registry (ranging from 76.4% to 88.0%) than with the Healthcare Registration File (ranging from 72.3% to 77.4%).

Table 3 presents the proportion of available perinatal and demographic variables in the Birth Registry between 1961 and 1974 for the 2,694 records successfully linked between the electronic BCG Registry and the Birth Registry. The completeness of information on these variables was remarkably high from 1965 onwards, with the exception of the type of birth (singleton, twins or triplets), which was documented for only 3.2% of subjects born in 1974. This was the result of a logistical problem in the Birth Registry, later solved by importing data from Statistics Canada where this information was available for virtually all subjects.

**Table 3:** Proportion of perinatal and demographic variables present in the Birth Registry for linked subjects (N = 2,694) per birth year

**Birth Year**	**Number of subjects**	**Birth weight**	**Gestational age**	**Type of birth**	**Number of livebirths**	**Residential district**	**Father’s place of birth**	**Mother’s place of birth**	**Father’s age**	**Mother’s age**
**1961**	155	0	0	0	100	0	0	0	0	0
**1962**	158	0	0	1.3	100	0	1.3	1.3	1.3	1.3
**1963**	182	0	0	100	100	0	98.9	99.5	98.9	100
**1964**	203	0	0	100	100	0	99.0	100	0.5	0.5
**1965**	178	100	100	99.4	73.6	98.3	97.8	99.4	98.9	100
**1966**	209	100	100	100	68.4	99.5	98.6	99.5	98.6	99.5
**1967**	197	100	100	98.0	64.0	96.5	97.0	97.5	97.5	98.0
**1968**	195	100	100	99.0	64.1	99.0	99.5	98.0	99.5	99.5
**1969**	196	100	100	95.4	60.7	94.9	91.8	94.4	93.4	95.4
**1970**	209	99.5	99.5	100	100	99.5	97.6	99.0	98.4	99.0
**1971**	193	100	100	100	100	99.0	98.5	100	98.5	100
**1972**	194	100	100	99.5	99.5	99.0	96.4	98.5	96.9	99.5
**1973**	208	100	100	99.0	99.0	98.6	97.6	99.0	97.6	99.0
**1974**	217	100	100	3.2	98.6	97.2	99.1	99.5	98.6	99.5

## Discussion

### Data quality

In the present study, we have assessed and documented data accuracy and completeness of the electronic Québec BCG Registry. This provided us with an opportunity to correct some systematic errors that have occurred during the computerization process, although very high agreement was observed between paper listings and the final electronic BCG Registry. Very few records (0.8%) had discrepancies in more than 1 variable upon comparing computerized records to their corresponding vaccination certificates. For discrepancies observed in given name and sex, general algorithms could then be applied to remove assigned generic names and verify the sex variable in the electronic BCG Registry before conducting further linkages with administrative databases.

Only one study was previously carried out to validate the Québec BCG Registry, comparing paper listings to childhood vaccination booklets [13]. Based on 369 subjects with both sources of information, the overall agreement was 78% (kappa = 0.56). A noteworthy limitation is that the vaccination booklet cannot be considered as a gold standard, since it might not have been provided by parents every time a vaccination took place.

### Linkage with administrative databases

Our results demonstrate that linkage of the electronic BCG Registry to administrative databases towards establishing a retrospective cohort study is highly feasible. However, in the absence of a unique identifier, the linkage method and missing values in personal identifiers had an impact on linkage success across birth years.

Approximately 60% of the electronic BCG Registry sample was linked to both the Birth Registry and the Healthcare Registration File. Successful overall linkage rates were much lower with the latter due to the use of a deterministic method, which did not allow for flexibility with regards to the initial quality of data or presence of missing values. Thus, subjects with missing information on any of the following four variables - child’s surname, child’s given name, sex, and date of birth - could not be matched. These variables were considered as absolutely necessary for linkage, while father’s name was optional. Information on given name was missing from the BCG registry when the vaccine was received very shortly after birth, presumably before the child’s name was chosen by parents. For linkage purposes, subjects were selected if they had received the vaccine between 1970 and 1974. Hence, there were no missing given names among those born from 1961 to 1968, as these children were 2 to 14 years old at the time of vaccination. Given name was missing from the BCG Registry for few subjects (4%) born in 1969, for 19.6% of those born in 1970, and up to 29.2% among subjects born in 1974. This artefact, related to our selection criteria and the use of a deterministic method, could explain the lower linkage rates (ranging from 54% to 59%) with the Healthcare Registration File among those born after 1969. As expected, the probabilistic linkage method performed better, except for those born in the early 1960s, likely due to the lower quality of information from the Birth Registry at that time. The quality of information compiled in the Birth Registry is generally considered to be best from 1975 onwards, but had likely started to improve in prior years. This was evidenced by the highest linkage rate (87.2%) achieved with the Birth Registry for the 1974 birth year.

The non-negligible proportion (12.5%) of subjects linked solely with the Healthcare Registration File could be due to the fact that the Birth Registry contains information only on those born in Québec. One foreseeable situation is that a child born outside of Québec who subsequently immigrated to Québec would be present in the Healthcare Registration File and the BCG Registry, but definitely not in the Birth Registry.

### Use of the electronic BCG Registry for research purposes

Although BCG vaccination in Québec was discontinued in the 1970s except for selected high risk groups (Inuit and many on-reserve First Nations infants in higher tuberculosis incidence communities as well as health workers) [14,15], scientifically pertinent research hypotheses could be addressed using the information available in the BCG Vaccination Registry. Before its computerization in 2010, the Québec BCG Registry had been used in research on tuberculosis [13,16,17], leukaemia [18,19], type 1 diabetes [20], and asthma [21]. Although data from this registry can be useful in various epidemiological study designs, their most efficient utilization will be through linkage with existing provincial medical administrative health databases. Such methodology will enable studying intended and unintended effects of BCG vaccination in a population-based setting. Our research team is particularly interested in investigating the impact of early life non-specific stimulation of the immune function, as resulting from BCG vaccination, on the prevention or development of inflammatory and auto-immune diseases in childhood [22,23].

Depending on the specific research questions investigated in future studies, variables documented in the Birth Registry may be used to control for confounding. In this sense, our finding that this information is present from birth year 1965 onwards will be extremely useful when planning future studies.

A limitation worth mentioning is that adverse events following immunization were not routinely compiled in the BCG Vaccination Registry, unfortunately impeding population-based research on this pertinent issue.

In Québec, there are no unique personal identification numbers (PINs) for citizens to allow unambiguous linkage between public registries. A particular strength of our study was thus the ability to carry out linkage despite the absence of a unique common identifier. Instead, we relied on identification variables available in the BCG and Birth Registries, as well as in the Healthcare Registration File. We demonstrated the feasibility of this approach and suggested avenues to pursue, such as probabilistic as opposed to deterministic linkage, when using historical data coupled with administrative databases. For future epidemiological studies, linkage with provincial medical databases will allow obtaining data from physician billing claims, prescription drugs claims, and hospitalizations.

The present study was crucial to the future use of a largely unexploited and comprehensive vaccination dataset for research purposes. Moreover, documentation of the various steps undertaken towards validation and linkage of several databases will provide important methodological clues to researchers aiming at assembling similar research infrastructures in other settings.

## Conclusion

Our findings demonstrate that the electronic BCG Registry is highly complete and accurate, remarkably for variables related to BCG vaccination, and thus represents a valuable valid database. Linkage of this registry to administrative databases, in order to establish population-based epidemiological studies, was proven highly feasible despite the lack of a unique identifier. Such future studies will enable filling important knowledge gaps on the potential health effects of early life non-specific stimulation of the immune function, as resulting from BCG vaccination.

## Competing interests

The authors declare that they have no competing interests.

## Authors’ contributions

MCR was responsible for the conception and design of the study and analytical strategy, interpretation of the data, and critical revision of the manuscript. She is the principal investigator for this study. FC coordinated the study, supervised preliminary analyses, conducted final analyses, and critically revised the manuscript. JL verified data quality, conducted preliminary analyses, and revised the manuscript. MEP contributed to the conceptualization of the study and critical revision of the manuscript. MZ contributed to the conceptualization of the study, interpretation of the data, as well as preparation and critical revision of the manuscript. All authors read and approved the final manuscript.

## Pre-publication history

The pre-publication history for this paper can be accessed here:

http://www.biomedcentral.com/1472-6947/14/2/prepub
